# MOX Sensors for Authenticity Assessment and Adulteration Detection in Extra Virgin Olive Oil (EVOO)

**DOI:** 10.3390/s26010275

**Published:** 2026-01-01

**Authors:** Elisabetta Poeta, Estefanía Núñez-Carmona, Veronica Sberveglieri, Alejandro Bernal, Jesús Lozano, Ramiro Sánchez

**Affiliations:** 1 Department of Life Sciences, University of Modena and Reggio Emilia, Via J.F. Kennedy, 17/i, 42124 Reggio Emilia, RE, Italy; 2 Institute of Bioscience and Bioresources (CNR-IBBR), National Research Council URT Reggio Emilia, Via J.F. Kennedy, 17/i, 42124 Reggio Emilia, RE, Italy; 3 Nano Sensor System srl (NASYS), Via Alfonso Catalani, 9, 42124 Reggio Emilia, RE, Italy; 4 Industrial Engineering School, University of Extremadura, 06006 Badajoz, Spain; 5 Centro de Investigaciones Científicas y Tecnológicas de Extremadura (CICYTEX), 06007 Badajoz, Spain

**Keywords:** MOX sensors, food fraud, electronic nose, IoT applications, innovative technology, certified products

## Abstract

Food fraud, particularly in the olive oil sector, represents a pressing concern within the agri-food industry, with implications for consumer trust and product authenticity. Certified products like Protected Designation of Origin (PDO) Extra Virgin Olive Oil (EVOO) are premium products that undergo strict quality controls, must comply with specific production regulations, and generally have a higher market price. These characteristics make them particularly vulnerable to economically motivated adulteration. In this study, the adulteration of PDO EVOO with Olive Pomace Oil (POO) and Olive Oil (OO) was investigated through a combined analytical approach. A traditional technique, gas chromatography–mass spectrometry (GC-MS) combined with solid-phase microextraction (SPME), was employed alongside an innovative method based on an electronic nose equipped with metal oxide semiconductor (MOX) sensors. GC-MS analysis enabled the identification of characteristic volatile compounds, providing a detailed chemical fingerprint of the different oil samples. Concurrently, the MOX sensor array successfully detected variations in the volatile profiles released by the adulterated oils, demonstrating its potential as a rapid and cost-effective screening tool. The complementary use of both techniques highlighted the reliability of MOX sensors in differentiating authentic PDO EVOO from adulterated samples and underscored their applicability in routine quality control and fraud prevention strategies.

## 1. Introduction

Olive oil is a key component of the Mediterranean diet, globally appreciated for its nutritional value, distinctive sensory characteristics, and numerous health benefits [1]. Among the main European producing countries are Spain, Italy, and Greece, which together account for approximately 70% of global production. In Europe, the overall annual production of olive oil averages around 2 million tons, although it may fluctuate due to climatic conditions and agronomic factors [2].

In this study, three categories of olive oil were investigated: Extra Virgin Olive Oil (EVOO), Olive Oil (OO), and Olive Pomace Oil (POO). Volatile compounds were analyzed using gas chromatography–mass spectrometry (GC-MS) combined with headspace solid-phase microextraction (HS-SPME).

Among the various categories, EVOO represents the highest quality class. It is obtained exclusively through mechanical processes conducted at low temperatures, without the use of solvents or chemical treatments, thus preserving the phenolic fraction, fat-soluble vitamins (particularly vitamin E), and the volatile compounds responsible for its unique aromatic profile. These sensory and nutritional characteristics make EVOO highly valued not only from a gastronomic perspective but also in the prevention of various chronic and degenerative diseases [3].

Numerous scientific studies support its beneficial effects, including a reduced risk of cardiovascular diseases, anti-inflammatory properties, and modulation of oxidative responses, thanks to its high content of polyphenols and natural antioxidants [4,5]. Beyond its health-promoting properties, certification of origin and quality is a crucial aspect. Protected Designation of Origin (PDO) oils represent the most authentic expression of the link between product and territory [6]. The PDO certification guarantees compliance with strict specifications that regulate, among other aspects, the cultivars used, agronomic practices, harvesting methods, and processing techniques. These oils not only represent a cultural and gastronomic heritage but also play an important economic role for producing areas, contributing to the enhancement of local traditions and the protection of biodiversity. However, the prestige and high market value of PDO oils make them particularly vulnerable to fraud and adulteration [7,8]. Common fraudulent practices include blending with lower-quality oils, the addition of seed oils, or the use of oils from different harvest years or regions from those indicated on the label [9]. According to the International Olive Council (IOC), approximately 10% of olive oil traded globally shows non-compliance with label claims, with negative consequences for consumers, who risk purchasing inferior products at unjustified prices, and for honest producers, who suffer in terms of reputation and economic return [10].

Considering these critical issues, it is essential to develop authentication methods that are rapid, reliable, and accessible, capable of supporting official controls and providing greater guarantees to consumers. A thorough understanding of the compounds present in EVOO makes it possible to detect alterations resulting from degradation processes, as well as potential fraudulent adulterations [11,12,13]. The identification of the aromatic characteristics of EVOO can be performed either through sensory evaluation (panel test) or by analyzing its volatile compounds. Although the panel test is an official method, it presents several disadvantages: it is costly and time-consuming, and its results may be influenced by subjective factors, such as the training and individual sensitivity of the panelists [14]. Among instrumental techniques, gas chromatography–mass spectrometry (GC-MS) combined with headspace solid-phase microextraction (HS-SPME) stands out for its high accuracy in the qualitative and quantitative analysis of the volatile fraction. This approach enables the acquisition of a detailed chemical fingerprint, useful for identifying cultivar, geographical origin, and potential adulterations. However, GC-MS also presents certain practical limitations, including complex sample preparation, lengthy analysis times, high equipment costs, and the requirement for highly specialized technical personnel [15,16].

In addition to chromatographic approaches, optical spectroscopic techniques have also been successfully applied to the detection of extra virgin olive oil adulteration. In particular, Laser-Induced Breakdown Spectroscopy (LIBS) and UV–Vis–NIR absorption spectroscopy have demonstrated high accuracy in discriminating EVOO from adulterated samples by exploiting elemental and molecular spectral information. While these laboratory-based techniques provide excellent predictive performance, they typically rely on complex instrumentation and are less suited for rapid or on-site screening applications [17].

In this context, the electronic nose (e-nose) emerges as an innovative and complementary tool, capable of providing rapid, non-destructive analyses that can potentially be applied directly in the field [18,19]. Its operation is based on an array of chemical sensors, in this case metal oxide (MOX) sensors, which are sensitive to the presence of volatile compounds [20]. When these molecules interact with the sensitive surface of the MOX sensors, variations in electrical conductivity occur, which are processed to generate a characteristic olfactory fingerprint of the sample. Among the main advantages of MOX sensors are their rapid response, robustness, potential for miniaturization, and ease of integration into automated systems. Moreover, the electronic nose does not require complex pre-treatment steps and can provide real-time results. However, its main limitation lies in the inability to identify individual volatile compounds: the system is designed primarily for discrimination and classification tasks rather than for detailed chemical characterization [21].

The combined use of GC-MS and the electronic nose thus represents a promising approach for olive oil authentication, merging the analytical precision of GC-MS with the operational speed and flexibility of the electronic nose.

The main objectives of this study were to evaluate the discriminative capability of the electronic nose in classifying different types of olive oils (extra virgin olive oil, olive oil, and olive pomace oil), to compare the olfactory fingerprints generated by the MOX sensor system with the detailed chemical profiles obtained through GC-MS, and to explore the development of predictive models for rapid and non-destructive authentication of olive oils.

Protecting authenticity and enhancing the quality of olive oil, particularly those certified as PDO (Protected Designation of Origin), are strategic priorities for the agri-food industry and for consumer protection. The adoption of integrated analytical approaches, combining established techniques such as GC-MS with innovative tools like MOX sensor-based electronic noses, represents a decisive step toward a more modern, efficient, and sustainable control system, in line with market demands and current challenges related to food safety and transparency [22].

## 2. Materials and Methods

In this study, three types of oils and their respective mixtures were analyzed: certified Protected Designation of Origin Extra Virgin Olive Oil (EVOO), Olive Oil (OO), and Olive Pomace Oil (POO). All samples were purchased from retail outlets in the large-scale distribution sector and stored at room temperature, ranging between 20 and 25 °C, outside of refrigeration. For each biological replicate, three independent measurements were performed for both gas chromatography–mass spectrometry (GC–MS) and e-nose analyses. Prior to analysis, the order of sample measurements was randomized using a computer-generated sequence to avoid systematic bias. In addition, the position of samples within the thermostatic bath and on the e-nose measurement platform was varied across replicates to minimize potential position effects.

### 2.1. Experimental Design

The study was conducted using two complementary approaches. The first involved the characterization of volatile organic compounds (VOCs) present in EVOO, OO, and POO samples through GC–MS combined with solid-phase microextraction (SPME). The second approach focused on analyzing the volatile fraction, or olfactory fingerprint, of both pure samples and their mixtures using a device equipped with MOX sensors.

### 2.2. Sample Preparation and Characterization of Oil Samples

A total of 30 samples were analyzed using the electronic nose (e-nose), including Extra Virgin Olive Oil (EVOO), Olive Pomace Oil (POO), Olive Oil (OO), and their respective mixtures prepared with adulteration percentages of 10%, 30%, and 50%. In addition, 9 pure oil samples (EVOO, POO, OO) were analyzed using the GC-MS technique.

**Figure 1:**
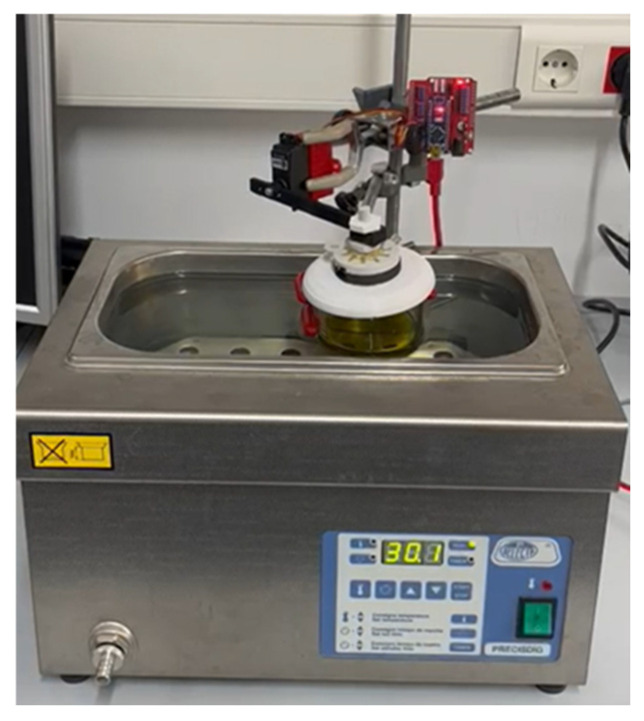
Analysis of oil samples using an electronic nose.

For the characterization of the volatilome, 50 mL of each pure sample was collected, transferred into glass containers, and subsequently analyzed using the electronic nose (e-nose). This volume was selected to ensure a stable and representative release of volatile compounds under dynamic headspace conditions and to maintain signal reproducibility over repeated acquisition cycles. Measurements were carried out in a thermostatic bath at 30 °C, a condition selected to promote the release of volatile compounds while minimizing oxidation or degradation processes in the sample (Figure 1).

In parallel, the same samples were analyzed using gas chromatography coupled with mass spectrometry (GC-MS), employing solid-phase microextraction (SPME) as the sampling technique. For this purpose, 20 mL of each sample were placed into sealed headspace vials, a volume optimized for the fixed vial geometry and liquid-to-headspace phase ratio required for reproducible HS-SPME analysis, sealed with caps featuring aluminum crimp tops and polyethylene-tetrafluoroethylene/silicone (PTFE/silicone) septa, ensuring tight sealing and preserving the integrity of the volatile profile.

The combined approach, integrating the rapid and non-destructive analysis of the e-nose with the detailed identification and quantification capabilities of GC-MS, allowed for a comprehensive and complementary characterization of the volatile fraction of each oil sample analyzed.

Several mixtures were subsequently prepared with the aim of evaluating the system’s sensitivity in detecting potential adulterations or combinations of different oils.

Specifically:

- 50% mixtures: three samples, obtained by combining 50 mL of POO with 50 mL of EVOO DOP; 50 mL of OO with 50 mL of EVOO DOP; and 50 mL of OO with 50 mL of POO.
- 30% mixtures: two samples, one consisting of 30 mL of POO and 70 mL of EVOO DOP, and the other of 30 mL of POO and 70 mL of OO.
- 10% mixtures: two samples, one containing 10 mL of POO and 90 mL of EVOO DOP, and the other 10 mL of POO and 90 mL of OO.

This experimental approach was designed to assess the ability of metal oxide sensors to detect even minimal variations in composition, simulating real-world adulteration scenarios and evaluating the system’s effectiveness in identifying fraud or quality alterations in olive oil. Table 1 provides a detailed description of the oil samples analyzed, including the number of replicates performed for each type.

### 2.3. Volatile Organic Compounds (VOCs)

The analysis of volatile organic compounds (VOCs) was conducted using two complementary approaches: (i) identification by gas chromatography coupled with mass spectrometry (GC-MS) using solid-phase microextraction (SPME), and (ii) olfactory profiling through an electronic nose system based on metal oxide semiconductor (MOX) sensors.

For the GC-MS analysis, two grams of oil were placed in hermetically sealed headspace vials and incubated at 40 °C for 10 min. A DVB/C-WR/PDMS SPME fiber (Supelco, Bellefonte, PA, USA) was then introduced into the vial and exposed in the headspace (4 cm) for 30 min under agitation. Subsequently, thermal desorption of volatile compounds was performed in the injector port at 240 °C for 5 min. Compound identification was carried out using a gas chromatograph (model 7820A; Agilent Technologies, Santa Clara, CA, USA) coupled to a mass spectrometer (5977E MSD; Agilent Technologies, Santa Clara, CA, USA). The initial oven temperature was set at 40 °C for 10 min, increased to 200 °C at 3 °C/min (held for 3 min), and finally raised to 240 °C at 10 °C/min, maintained for 5 min. Separation was achieved using an HP-5MS capillary column (30 m × 0.25 mm × 0.25 μm; Agilent J&W, Santa Clara, CA, USA) with helium as the carrier gas at a constant flow of 1 mL/min. Injections were performed in splitless mode at 240 °C, with the transfer line maintained at 250 °C and the ion source at 230 °C. Electron ionization energy was set at 70 eV, and data acquisition was performed in scan mode from 30 *m*/*z* to 300 *m*/*z*. Qualitative analysis was based on the comparison of the obtained mass spectra with reference spectra available in the NIST Mass Spectral Search Program (v2.0; National Institute of Standards and Technology (NIST), Gaithersburg, MD, USA). This methodology enabled the identification of representative compounds present in the oils, including aldehydes, alcohols, acids, and esters, allowing a precise differentiation of volatilome composition across pomace, virgin, and extra virgin oils, as well as the detection of potential adulteration.

In parallel, a portable electronic nose system based on MOX sensors was employed. This compact device is designed for the rapid detection of volatile profiles by recording impedance changes in the sensing elements upon exposure to VOCs released by the samples. Analyses were conducted at 30 °C using a thermostatic bath, ensuring headspace generation without inducing thermal degradation of the oils. The system captured subtle variations in the volatile profiles, generating characteristic olfactory fingerprints that allowed reliable classification of the samples. The electronic nose integrates a multichannel MOX sensor with four resistive sensing elements, each displaying differential sensitivity to specific chemical families, including alcohols, aldehydes, ketones, and fatty acids. Operating at a controlled working temperature of approximately 400 °C, the sensor ensured stable and reproducible responses. The embedded system incorporates data acquisition electronics and wireless connectivity, facilitating its application as a portable and compact analytical tool. The recorded signals, based on impedance variations of each sensing element, provided unique olfactory patterns for each sample, enabling a robust and non-destructive characterization of the oils.

### 2.4. Electronic Nose Set-Up

**Figure 2:**
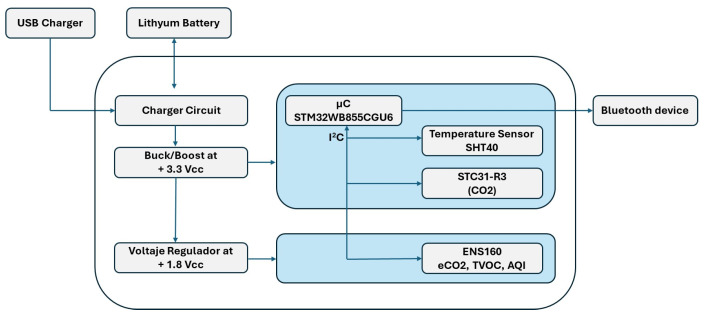
Block diagram of the device.

The printed circuit board (PCB) of the system integrates various electronic components, as shown in Figure 2. The board is powered via a USB port and a 3.7 V lithium battery. The battery voltage is regulated by a buck–boost converter, which stabilizes the output at 3.3 V, supplying most of the electronic components. In addition, a linear voltage regulator further reduces this voltage to 1.8 V to power the ENS160 sensor (ScioSense, Eindhoven, The Netherlands).

The data acquisition system is based on three different sensors, whose characteristics and measured signals are summarized in Table 2. The sensor outputs are transmitted via the I2C protocol to a 32-bit microcontroller (STM32WB55CGU6, STMicroelectronics, Geneva, Switzerland). This microcontroller manages both sensor communication and wireless data transmission via Bluetooth Low Energy (BLE) to external devices, such as smartphones, enabled by the integrated Arm Cortex-M0+ core (Arm Ltd., Cambridge, United Kingdom). Similar electronic nose architectures, combining low-power sensor interfaces, embedded microcontrollers, and wireless communication modules, have been successfully employed for the detection and discrimination of volatile organic compounds, as reported in the literature [23].

The sensor array integrated in the portable e-nose comprises an ENS160 MOX gas sensor (TVOC/eCO_2_/AQI outputs) an SHT40 (temperature/relative humidity sensor; Sensirion AG, Stäfa, Switzerland), and an STC31-R3 (CO_2_ sensor; Sensirion AG, Stäfa, Switzerland) (see Table 2 for details).

Temperature and relative humidity were continuously recorded (SHT40) as quality-control/reference variables to confirm stable measurement conditions and were not included as input features in the PCA/ML models.

**Figure 3:**
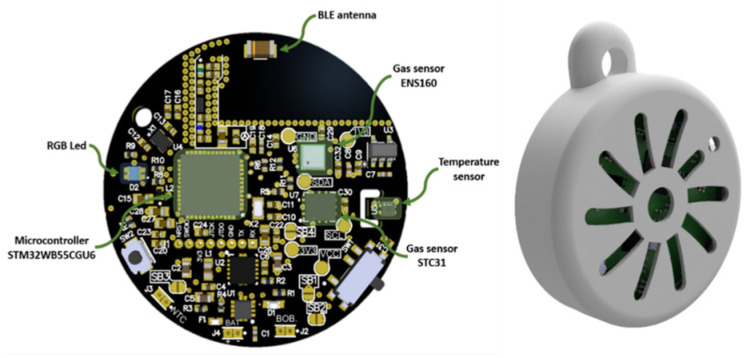
Electronic board (**left**) and case (**right**).

On one hand, Figure 3 (left) shows the layout of the printed circuit board (PCB), designed in a compact circular format with a diameter of 36.4 mm. The components have been strategically placed to maximize space efficiency. Among the key elements are a microcontroller, responsible for managing sensor communication and Bluetooth Low Energy (BLE) connectivity, and an RGB LED used to visually indicate air quality levels.

The sensing system includes three main sensors: the ENS160 gas sensor for air quality evaluation, the STC31 gas sensor for CO_2_ concentration measurement, and a dedicated temperature, along with a dedicated temperature and relative humidity sensor (SHT40). Communication between the sensors and the microcontroller is handled via the I2C protocol. A BLE antenna is also integrated to allow wireless data transmission to external devices such as smartphones.

Figure 3 (right) presents the external casing that encloses the PCB. This protective structure is designed with radial vents that enable air to circulate freely to the terminal sensors. The enclosure is optimized for portability and comfortable everyday use, ensuring that environmental measurements can be carried out reliably while the device is worn or transported.

## 3. Results and Discussion

### 3.1. Characterization and Comparison of the Volatile Components of Pure Oils Using SPME-GC-MS

The analysis of the volatile profile of the three oils under investigation (EVOO, OO, POO), performed using gas chromatography–mass spectrometry (GC-MS) coupled with solid-phase microextraction (SPME), enabled the identification of 60 compounds. These were classified into seven main chemical groups: alkanes, alkenes, carboxylic acids, aldehydes, alcohols, ketones, and others (Table 3).

**Figure 4:**
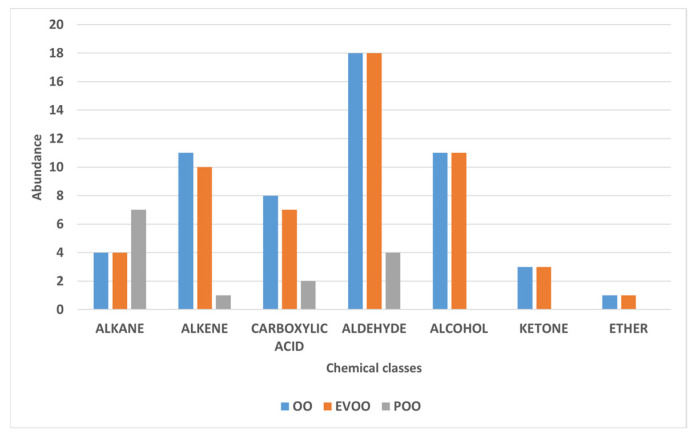
Chemical classes of Extra Virgin Olive Oil (EVOO), Virgin Olive Oil (OO), and Olive Pomace Oil (POO) samples analyzed by gas chromatography–mass spectrometry (GC–MS).

The Extra Virgin Olive Oil (EVOO) samples exhibited a rich and complex aromatic profile, characterized by a high abundance of volatile compounds belonging to the C6 family, including aldehydes (hexanal, E-2-hexenal), alcohols (1-hexanol, (Z)-3-hexen-1-ol), and their corresponding esters, such as (Z)-3-hexen-1-yl acetate. These compounds are primarily derived from the enzymatic lipoxygenase (LOX) pathway, which is activated during the processing of fresh and intact olives, converting polyunsaturated fatty acids into volatile metabolites with low odor thresholds. These molecules are responsible for the fresh, green, fruity, and herbaceous sensory notes that are typical of high-quality olive oils. Hexanal is one of the most abundant compounds in the volatile profile of EVOO and significantly contributes to green and freshly cut grass olfactory sensations, as widely reported in the literature [72,73,74]. E-2-hexenal, which is also frequently detected at high concentrations, is associated with a more intense and penetrating fruity aroma. According to García-Vico et al. (2017) [75], this compound is often the most abundant among the C6 volatile organic compounds (VOCs). C6 alcohols, such as 1-hexanol and (Z)-3-hexen-1-ol, also contribute to the aromatic profile by imparting sweet, floral, and slightly fruity notes. In particular, the latter serves as an important precursor of volatile esters such as (Z)-3-hexen-1-yl acetate, which imparts fresh and fruity aromatic nuances (e.g., tomato leaf, green banana), significantly enhancing the overall olfactory complexity of the oil [18]. Overall, aldehydes, alcohols, and their corresponding esters are the main contributors to the aromatic characteristics of EVOO, not only due to their abundance but also because of their low sensory threshold and high sensory impact (Figure 4). Their presence at high concentrations is commonly associated with high-quality raw material and a prompt, well-executed extraction process, thus representing reliable markers of the product’s freshness and integrity [75,76,77].

**Figure 5:**
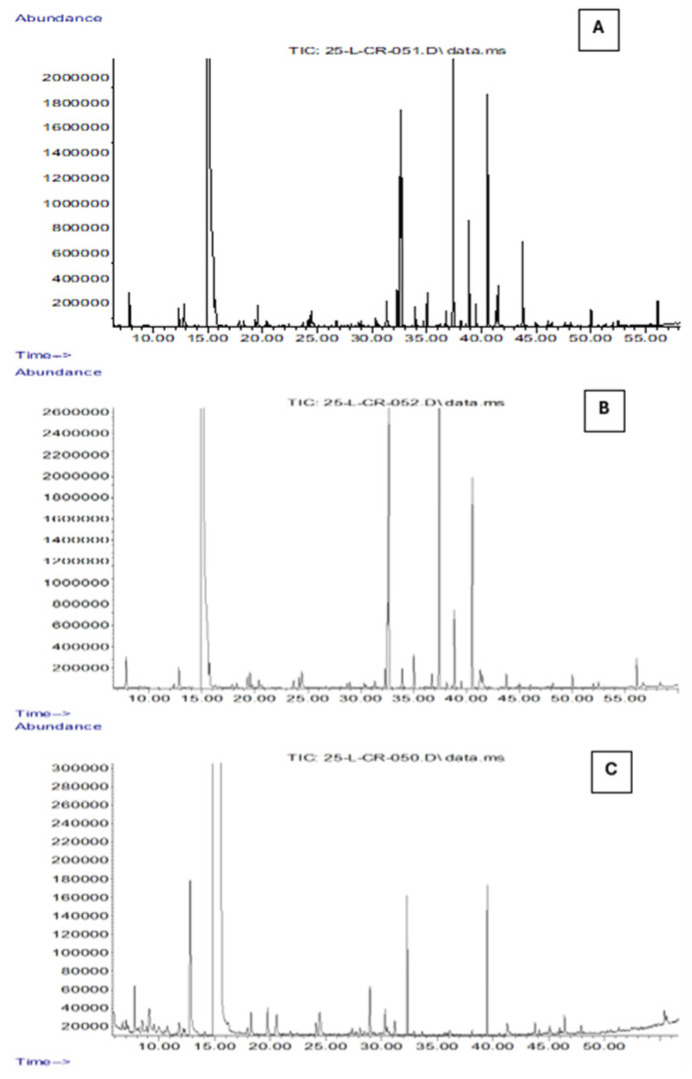
Total ion chromatograms (TICs) obtained by GC-MS analysis coupled with SPME of OO (**A**), EVOO (**B**), and POO (**C**) samples. The x-axis represents the retention time (min), while the y-axis indicates the relative abundance of the detected volatile compounds.

Although Virgin Olive Oil (OO) shares many volatile compounds with Extra Virgin Olive Oil (EVOO), it is characterized by a less intense and less complex aromatic profile, indicative of lower commercial and sensory quality (Figure 5). The reduced presence of key volatile compounds in OO samples suggests a weakening or partial disruption of the main biosynthetic pathways. This is often associated with the use of lower-grade raw materials, longer storage times, or technological treatments such as refining [78].

**Figure 6:**
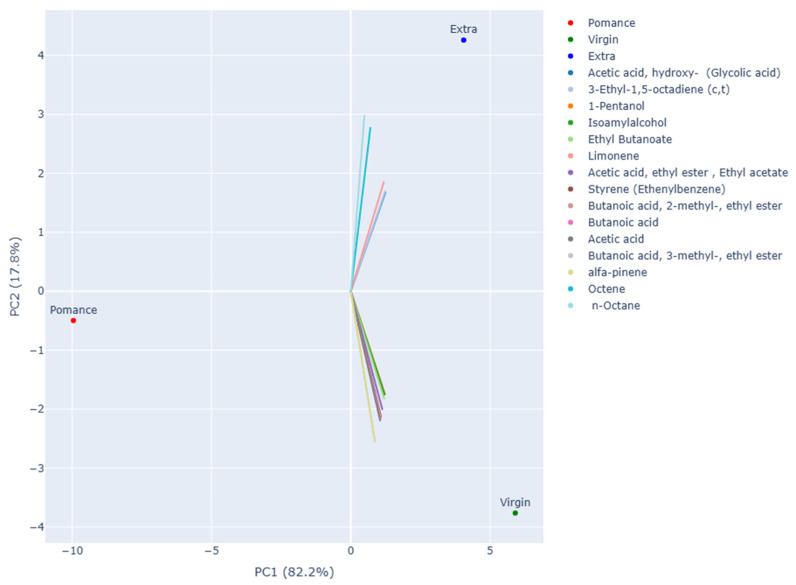
Interactive 2D PCA plot based on volatile compound data, displaying the 15 most influential loading vectors contributing to sample separation.

Principal Component Analysis (PCA) was applied to the volatile compound data obtained by gas chromatography–mass spectrometry (GC–MS) in order to investigate differences among the various types of olive oil analyzed. The resulting two-dimensional projection (Figure 6) shows that the first two principal components account for the total variability of the dataset: PC1 alone explains 82.2% of the variance, while PC2 accounts for 17.8%.

The distribution of the samples along PC1 highlights a clear separation between olive pomace oil and the other two groups. Pomace samples cluster at the far left of the plot, confirming a distinct aromatic profile characterized by the absence of key compounds and the presence of metabolites associated with oxidative and degradative processes.

The second principal component allows for a clear distinction between Extra Virgin Olive Oil (EVOO) and virgin olive oil (VOO). EVOO samples group in the upper right quadrant of the plot, suggesting a greater abundance of compounds derived from the enzymatic lipoxygenase (LOX) pathway, typically responsible for fresh and green sensory notes. Conversely, virgin olive oil samples are located in the lower right quadrant, showing an intermediate profile—closer to EVOO than to olive pomace oil—but with quantitative differences in the concentration of the most representative volatiles.

The loading analysis supports these observations: alcohols (e.g., 1-pentanol, isoamyl alcohol), esters (ethyl butanoate, ethyl acetate, esters of butanoic acid), and terpenes (such as limonene and α-pinene) are strongly associated with EVOO, contributing to its distinctive aromatic fingerprint. In contrast, short-chain acids (acetic acid, butanoic acid) and degradation-related molecules (octene, n-octane, styrene) appear more representative of pomace samples, and to a lesser extent of virgin olive oils. Overall, PCA effectively highlighted marked differences among the three olive oil categories, confirming that the volatile profile represents a reliable indicator of both product quality and preservation status.

### 3.2. Measurement Setup

The results obtained with GC-MS were compared with those acquired using the electronic nose, followed by a PCA to explore group separations and classification capabilities. Similar approaches are widely reported in the literature, where electronic noses, often combined with multivariate statistical tools, have been successfully applied to the discrimination of olive oils according to geographical origin, cultivar, and quality grade, yielding results comparable to those of consolidated analytical platforms such as GC-MS [17,18].

In the present study, measurements on olive oil samples were performed using a dynamic headspace configuration based on cyclic adsorption and desorption, specifically designed to capture the temporal evolution of volatile organic compounds (VOCs). The setup consisted of a cylindrical glass chamber with an internal volume of approximately 265 mL (75 mm diameter × 600 mm height). Pure and blended olive oil samples were introduced in fixed aliquots of 100 mL to ensure experimental reproducibility. The sensing unit was positioned directly above the headspace to maximize exposure to the emitted volatiles.

Each measurement session comprised 18 automated adsorption/desorption cycles. During the adsorption phase (60 s), the sensor was exposed to the headspace, allowing VOCs to accumulate on the sensing surface; during the subsequent desorption phase (60 s), the chamber was flushed with ambient air to restore baseline conditions and minimize hysteresis. This cyclic protocol enabled the acquisition of transient response dynamics while avoiding sensor saturation, a feature that distinguishes it from static headspace measurements commonly adopted in other e-nose studies.

The ENS160 gas sensor, integrated into a custom portable device, provided real-time TVOC measurements (ppb) over 18 consecutive cycles, generating robust time-series datasets for each sample. This acquisition strategy improved signal clarity and reproducibility and enabled advanced data processing approaches, including temporal pattern recognition and the training of machine learning models (e.g., neural networks). Compared with previous studies primarily based on steady-state signals or averaged responses, the proposed cyclic approach provides greater analytical depth and supports the development of portable, cost-effective, and data-driven alternatives to conventional laboratory-based GC-MS analysis.

#### Temporal Response of the Sensors

**Figure 7:**
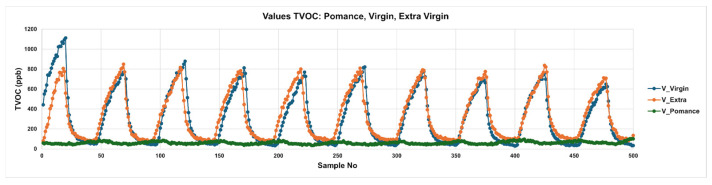
Temporal TVOC Response Profiles for Pure Olive Oil Samples: Extra Virgin, Virgin, and Pomace.

Figure 7 displays the temporal evolution of total volatile organic compounds (TVOCs) for three pure olive oil categories: extra virgin (V_Extra), virgin (V_Virgin), and pomace (V_pomace). The plotted curves represent the TVOC response over time during alternating adsorption and desorption phases. Extra virgin and virgin oils exhibit distinct and reproducible peak patterns, characterized by sharp rises during the adsorption phases followed by gradual descents in the desorption phases. Notably, extra virgin oil consistently produces the highest TVOC amplitudes, while virgin oil follows a slightly lower profile. In contrast, olive pomace oil shows a markedly flattened response, with minimal peak formation and subdued VOC release. This behavior reflects the reduced content of aroma-active volatiles in lower-quality oils, consistent with their known sensory degradation.

**Figure 8:**
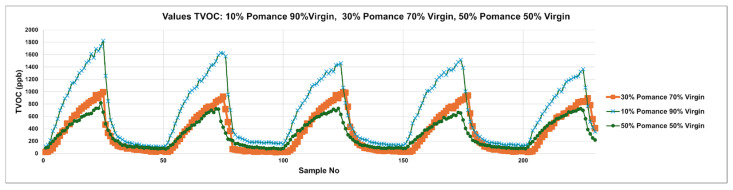
Temporal TVOC Response Profiles for Adulterated Olive Oils: Virgin Blends with 10%, 30%, and 50% Pomace.

Figure 8 extends the analysis to blended samples created by mixing virgin olive oil with 10%, 30%, and 50% olive pomace oil. The cyclic TVOC patterns reveal a clear gradient in signal amplitude and dynamics associated with the degree of adulteration. The 10% blend (blue curve) maintains a peak structure similar to that of pure virgin oil but with slightly diminished intensities. As pomace content increases to 30% (orange curve) and 50% (green curve), the peaks become broader and less pronounced, indicating significant disruption of the volatile profile. These shifts in amplitude and temporal shape confirm the system’s sensitivity to adulteration, even at low levels, and highlight the diagnostic potential of MOX sensor arrays for real-time detection of olive oil authenticity.

### 3.3. Discrimination of Oil Samples Using a MOX Sensor-Based Device

**Figure 9:**
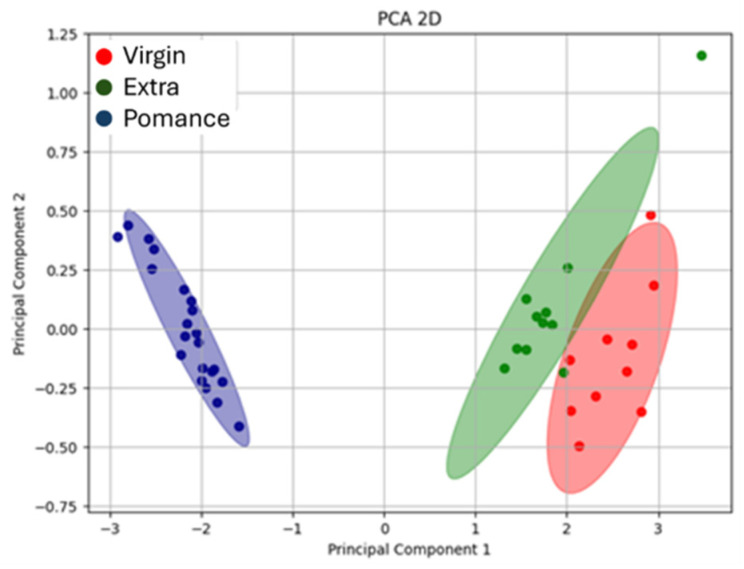
Three-dimensional Principal Component Analysis (PCA) showing the separation between extra virgin olive oil (green), olive pomace oil (blue), and virgin olive oil (red) samples.

The use of Principal Component Analysis (PCA) proved to be a particularly effective approach for exploring and visualizing the discriminative capability of the electronic nose based on MOX sensors in recognizing different types of oil and their mixtures. Initially, all available sensor outputs were considered; however, channels that added noise or did not improve discrimination/model performance were excluded from the final feature set to avoid unnecessary dimensionality. The plot shown (Figure 9) displays the projection of the data obtained from the electronic nose responses along the first principal components, which account for most of the variability within the dataset. This MOX sensor-based analysis was applied to samples of extra virgin olive oil, olive pomace oil, and virgin olive oil, with the aim of highlighting potential differences in the volatile compound profiles among the various oil types.

**Figure 10:**
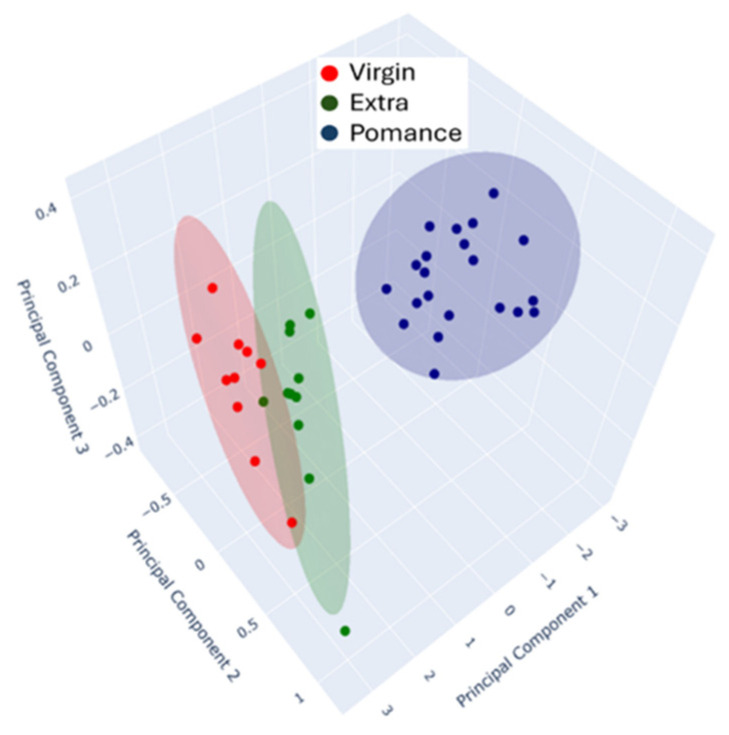
3D Principal Component Analysis (PCA) of MOX sensor responses for olive oil classification.

To complement the two-dimensional projection, a three-dimensional PCA plot was also generated (Figure 10), offering a more detailed visualization of the clustering patterns among the oil samples. This 3D representation allows for a better assessment of the group separation and distribution in multidimensional space.

In this case, Principal Components 1, 2 and 3 explain 97.32%, 2.25%, and 0.43% of the total variance, respectively, indicating that most of the variability in the dataset is captured by the first component alone. Despite the relatively low contribution of PC2 and PC3, their inclusion enhances the spatial perception of class separability and emphasizes subtle differences among samples, particularly within mixtures and borderline cases.

Together, both PCA plots (Figure 8 and Figure 9) illustrate the strong discriminative power of the MOX-based electronic nose in differentiating olive oil categories. The high variance explained by the first component in both the 2D and 3D analyses demonstrates the consistency and reliability of the sensor responses in capturing the key features that distinguish pomace, virgin, and extra virgin oils.

**Figure 11:**
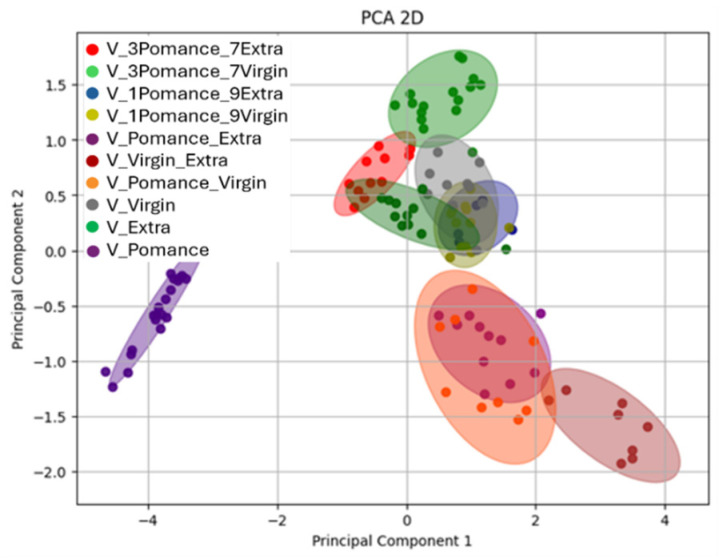
Two-dimensional Principal Component Analysis (PCA) demonstrating the separation between pure and blended oil samples in various proportions. Colors indicate the different combinations analyzed: blue for 10% olive pomace oil + 90% extra virgin olive oil, olive-green for 10% olive pomace oil + 90% virgin olive oil, red for 30% olive pomace oil + 70% extra virgin olive oil, light green for 30% olive pomace oil + 70% virgin olive oil, green for pure extra virgin olive oil, purple for pure olive pomace oil, brown for 50% olive pomace oil + 50% extra virgin olive oil, yellow for 50% olive pomace oil + 50% virgin olive oil, gray for pure virgin olive oil, and green for extra virgin olive oil.

To further explore the potential of the MOX-based electronic nose to detect and classify adulterated olive oils, an additional Principal Component Analysis (PCA) was performed on a dataset including both pure samples and blends in various proportions. Figure 11 presents the two-dimensional PCA projection, where each point corresponds to a sample measurement, and color-coded ellipses represent the different blend combinations and pure categories.

The analysis reveals a well-defined separation between pure oils (extra virgin, virgin, and pomace) and their respective mixtures. The distribution of the samples in the PC1-PC2 plane reflects a gradient in volatile profile composition according to blend ratios. Notably, Principal Component 1 (PC1) explains 82.04% of the total variance, while PC2 accounts for 16.15%, providing a comprehensive representation of the variability within the dataset. The pure extra virgin olive oil samples (green) appear tightly grouped and are clearly distinct from both pomace (purple) and virgin oils (gray).

Blended samples form intermediate clusters depending on the ratio and type of oils used. For instance, the red cluster (30% pomace + 70% extra) and light green cluster (30% pomace + 70% virgin) are located closer to the extra virgin and virgin oil clusters, respectively. The blue and olive-green cluster (10% pomace + 90% extra / virgin) are more centrally positioned, while the light purple, orange, and brown clusters (50% blends) are placed further away from the pure groups, indicating greater divergence in the volatile profiles. The confidence ellipses illustrate good internal consistency within each group and limited overlap, confirming the system’s sensitivity to subtle changes in composition.

**Figure 12:**
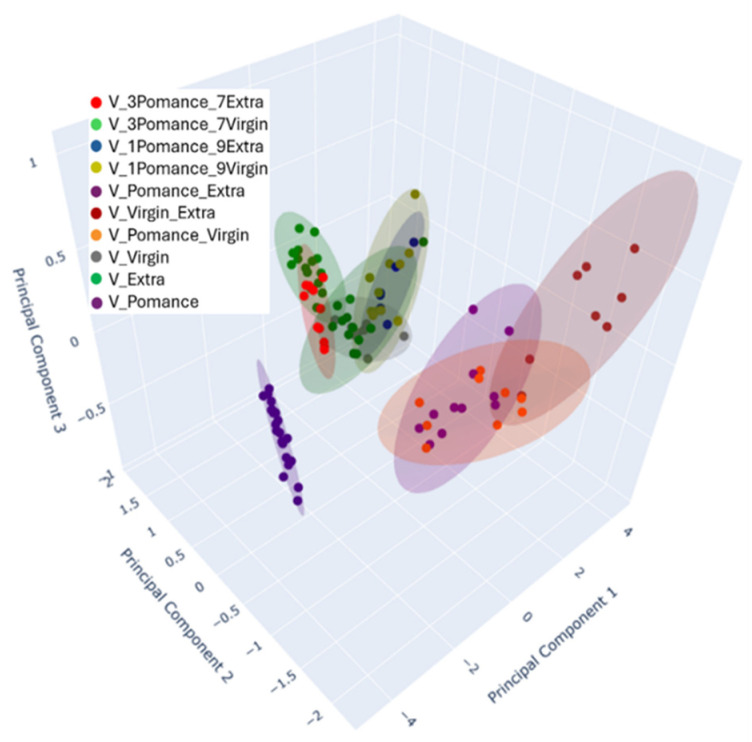
Three-dimensional Principal Component Analysis (PCA) demonstrating the separation between pure and blended oil samples in various proportions. Colors indicate the different combinations analyzed: blue for 10% olive pomace oil + 90% extra virgin olive oil, olive-green for 10% olive pomace oil + 90% virgin olive oil, red for 30% olive pomace oil + 70% extra virgin olive oil, light green for 30% olive pomace oil + 70% virgin olive oil, green for pure extra virgin olive oil, purple for pure olive pomace oil, brown for 50% olive pomace oil + 50% extra virgin olive oil, yellow for 50% olive pomace oil + 50% virgin olive oil, gray for pure virgin olive oil, and green for extra virgin olive oil.

To provide a more nuanced visualization of these complex relationships, a three-dimensional PCA plot was constructed (Figure 12). The inclusion of Principal Component 3 (PC3), which accounts for 1.09% of the variance, enriches the interpretation by revealing spatial patterns and separations not evident in the 2D representation. The 3D plot maintains the separation among pure and blended oils, further reinforcing the electronic nose’s capability to distinguish not only between different oil categories but also between subtle blend proportions.

### 3.4. Multilayer Perceptron Analysis

**Figure 13:**
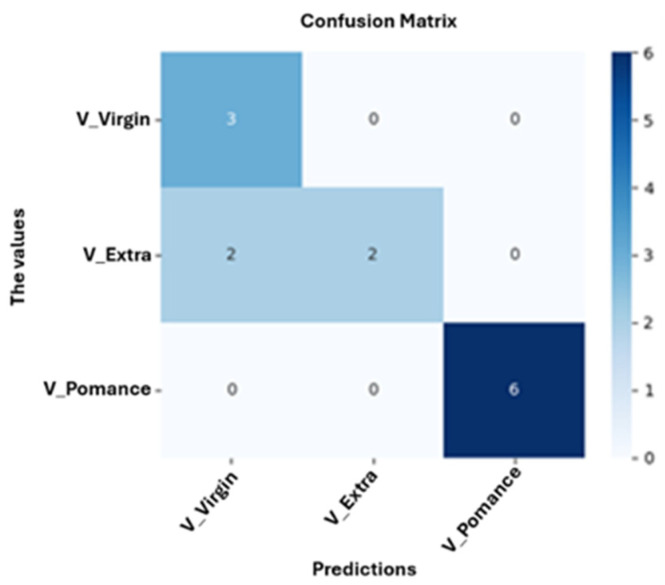
Confusion Matrix for Classification of Pure Olive Oil Categories: Virgin, Extra Virgin, and Pomace.

Figure 13 shows the confusion matrix obtained for the neuronal network model trained to classify olive oils into three categories: pomace, virgin, and extra virgin. The model achieved an overall accuracy of 84.62%, with perfect classification of olive pomace oil, partial misclassification of extra virgin oils as virgin, and correct classification of virgin samples. These results suggest that while the model robustly identifies olive pomace oil, the classification between extra virgin and virgin oils is more challenging due to their compositional similarity.

**Figure 14:**
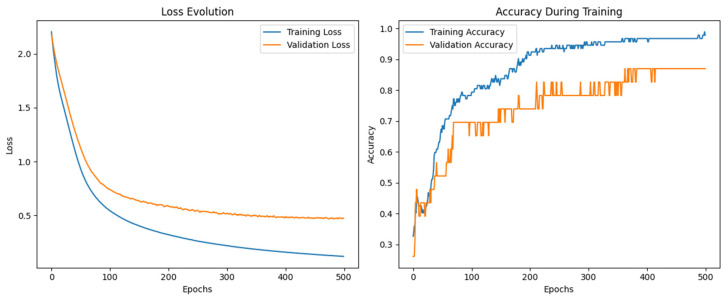
Neuronal network performance for classifying pure olive oils.

Figure 14 displays the evolution of the loss and accuracy during training. As shown in the loss curve, both training and validation losses decrease significantly over the epochs, with the training loss reaching values near 0.2 after 500 epochs. In parallel, training and validation accuracy gradually increases, stabilizing above 90% in the final stages, confirming the convergence and generalization capability of the model.

**Figure 15:**
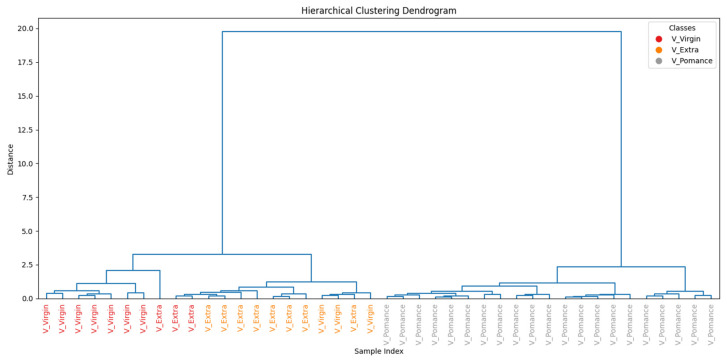
Hierarchical clustering of pure olive oil categories based on TVOC profiles.

To further assess the similarity among the oil samples, Figure 15 presents the hierarchical clustering dendrogram. A clear separation between pomace and the other two categories is observed, supporting the findings from PCA and classification results. Extra virgin and virgin oils form distinct subclusters, but with closer proximity, reflecting their overlapping volatile profile.

**Figure 16:**
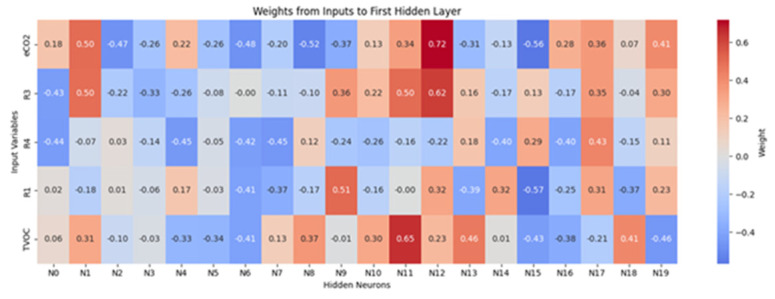
Connection weights from input variables to the first hidden layer neurons.

**Figure 17:**
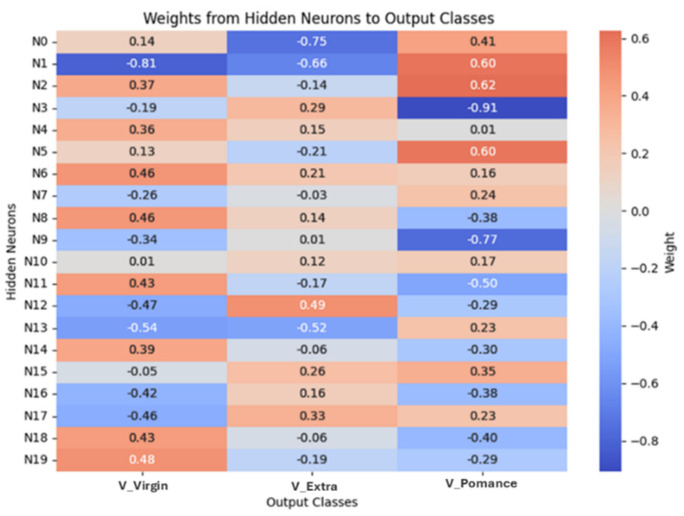
Connection weights from hidden layer neurons to output classes.

Figure 16 and Figure 17 report the learned connection-weight matrices of the MLP. These matrices are provided for transparency as a qualitative visualization of the trained parameters. Because multilayer neural networks are non-linear and predictors may be correlated, individual weights are not directly interpretable as reliable feature importance. Therefore, we do not draw feature-importance conclusions from these plots, and model assessment is based on the classification performance reported in the Results.

**Figure 18:**
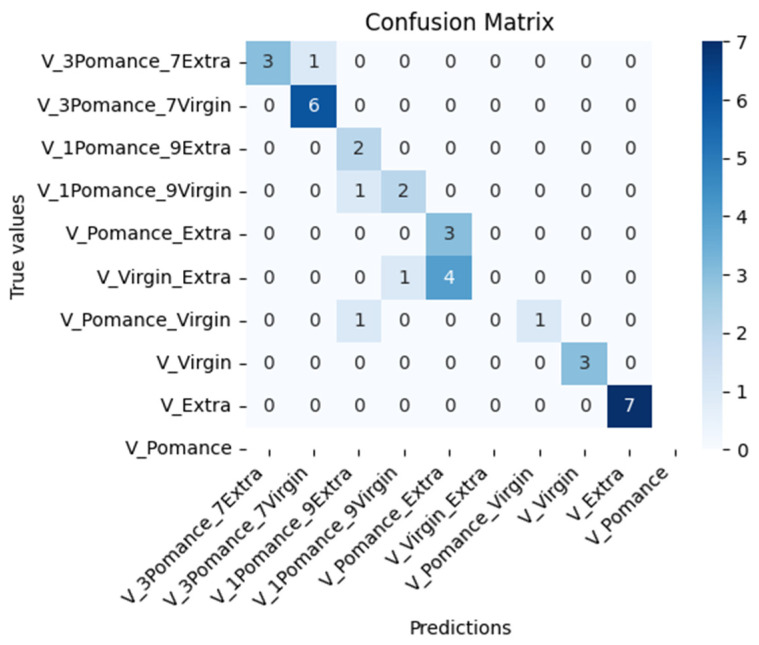
Confusion matrix for classification of pure and adulterated olive oils.

Figure 18 shows the confusion matrix corresponding to the classification of pure and adulterated olive oil samples using the MOX-based electronic nose. The model was trained with 500 epochs and includes 10 distinct classes: pure extra virgin, virgin, and olive pomace oil, as well as binary mixtures adulterated with 10%, 30%, and 50% olive pomace oil. The model achieved an overall accuracy of 77.14%, correctly classifying most pure samples (e.g., extra virgin and pomace) and several of the blended classes. Misclassification primarily occurred among blends with low pomace content (e.g., 10% or 30%), likely due to their similar volatile profiles.

Although formal LOD/LOQ values are not directly applicable to this electronic-nose pattern-recognition method, the lowest adulteration level tested in this work was 10% (*v*/*v*), at which discrimination of pure oils was achieved under controlled conditions. Determining a formal limit of detection/quantification will require further experiments at levels below 10%, with greater replication and an explicit decision criterion.

**Figure 19:**
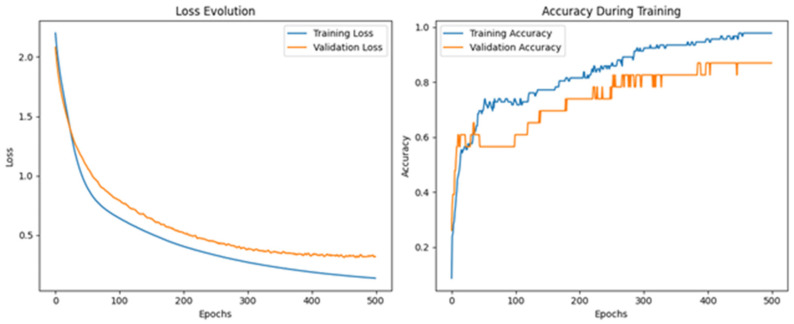
Training and validation performance of the neural network for olive oil adulteration detection.

Figure 19 displays the evolution of the training and validation loss (left) and accuracy (right) over the course of 500 training epochs. As observed, both training and validation loss steadily decrease with increasing epochs, while the accuracy curves show a gradual increase and stabilization beyond 400 epochs. The model converges with training accuracy close to 99% and validation accuracy stabilizing around 87% indicating good generalization performance despite the increased number of output classes.

**Figure 20:**
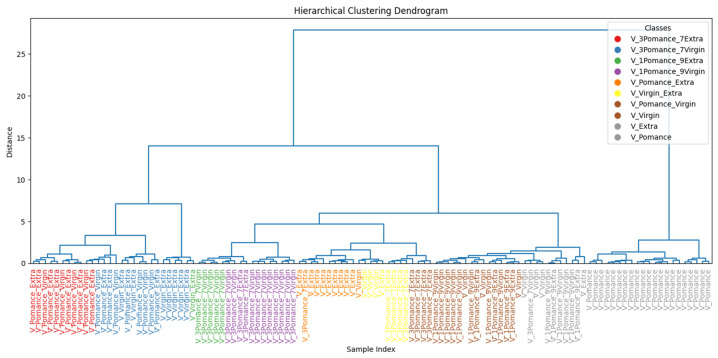
Hierarchical clustering of pure and adulterated olive oil samples based on sensor response.

To better understand sample similarities, Figure 20 shows a hierarchical clustering dendrogram generated from the learned features. The pure oil samples form well-defined and coherent clusters, while blended samples are grouped in intermediate positions, reflecting their compositional nature. Notably, mixtures with 50% pomace (such as V_Pomace_Extra and V_Pomace_Virgin) are positioned closer to the pomace cluster, suggesting that even moderate adulteration levels significantly alter the volatile profile.

**Figure 21:**
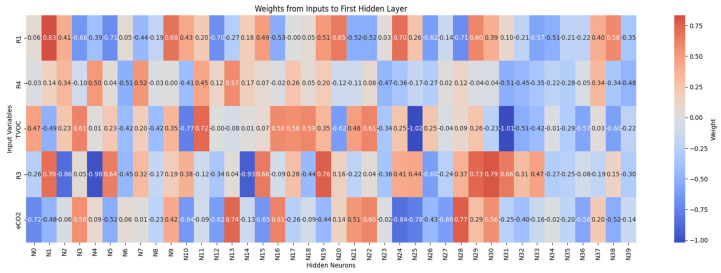
Synaptic weights from input features to neurons in the first hidden layer (deep network).

**Figure 22:**
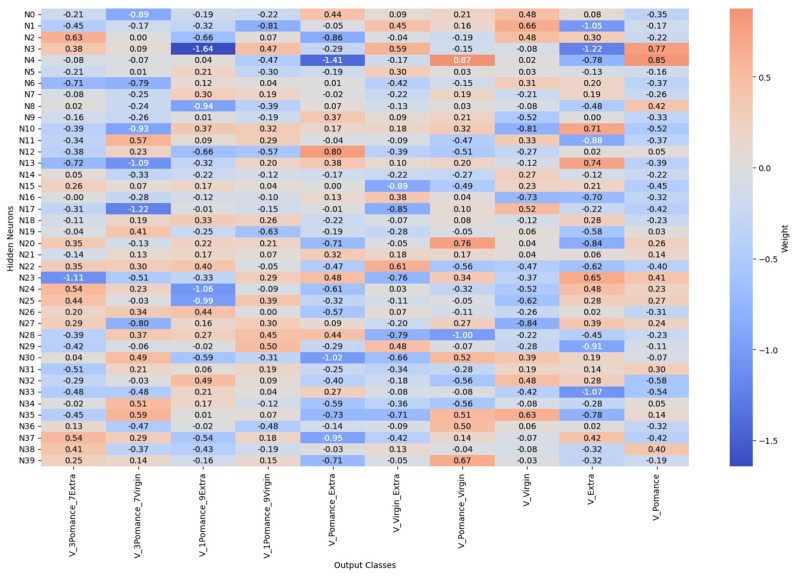
Synaptic weights from hidden neurons to output classes in extended olive oil classification.

Figure 21 and Figure 22 show the weight matrices of the fully connected layers in the extended model. They are included as a qualitative depiction of the learned parameterization and should be interpreted cautiously; they do not constitute a formal measure of variable importance. More rigorous interpretability analyses will be considered as future work to quantify input influence under controlled validation conditions.

## 4. Conclusions

The results obtained confirm the effectiveness of the electronic nose in distinguishing between different types of olive oil, demonstrating its ability to detect significant variations in volatile profiles. The comparison with GC-MS data revealed a clear correspondence between the olfactory fingerprints provided by the MOX-based sensor array and the chemical composition of the samples. In particular, GC-MS analysis indicated that differences among oil categories were mainly associated with variations in key classes of volatile compounds, such as aldehydes, alcohols, esters, and ketones, which are known to play a major role in defining olive oil aroma and quality and are sensitive to processing conditions and adulteration.

These compound groups, rather than individual markers, contribute collectively to the recognition of oil authenticity and are effectively captured by the pattern-based response of the electronic nose. The integration of GC-MS with multivariate chemometric analysis, including PCA, therefore provides complementary information, combining detailed chemical insight with rapid discrimination capability. This integrated approach reinforces the potential of the MOX-based electronic nose as a fast, non-destructive screening tool for olive oil quality control and authenticity assessment, particularly for certified products such as PDO olive oils.

It is important to note that MOX sensors exhibit cross-sensitivity, meaning that their response is not specific to a single compound but reflects the combined effect of different VOC families (and may also be influenced by temperature and humidity). However, this behavior is consistent with the electronic-nose concept, which aims to mimic human olfaction: similarly to a sensory panel that perceives the global aroma profile rather than isolated compounds (as in chromatography), the e-nose captures an overall volatile fingerprint of the sample. Therefore, discrimination is not based on single-sensor selectivity, but on correlating multichannel signals and modeling them using multivariate methods (e.g., PCA) and machine-learning algorithms to extract robust patterns related to authenticity and adulteration.

Overall, the adoption of complementary analytical techniques represents a promising strategy to address current challenges in olive oil authentication, supporting the development of more efficient, sustainable, and transparent control systems. Future work will focus on expanding the dataset, refining predictive models, and implementing real-time and in-line monitoring solutions, with the aim of facilitating the integration of MOX-based sensing technologies into industrial production chains. Moreover, in future work, IoT integration of the system will require addressing practical implementation challenges: (i) ensuring robust data acquisition and transmission (e.g., BLE/Wi-Fi) by incorporating local storage and delayed retransmission in case of connectivity losses; (ii) optimizing power consumption through sleep modes and measurement duty-cycling (periodic sampling vs. continuous monitoring) to ensure sufficient autonomy; (iii) defining the model deployment strategy (on-device or near-edge ‘gateway’ inference vs. cloud inference), balancing latency, privacy, and cost; (iv) ensuring stable performance in real environments through temperature/humidity compensation, control of background VOCs, and drift/recalibration strategies; and (v) establishing traceability and maintenance of the model over time (model versioning and controlled model updates).

## Figures and Tables

**Table 1:** Schematic representation of the analyzed oil samples, indicating the number of replicates performed, the analytical techniques employed, and the total number of samples subjected to analysis.

Sample	Replicate (R)	Techniques	Sample Number
EVOO (20 mL for GC-MS); EVVO (50 mL for e-nose)	R1	GC-MS SPME; e-nose	1
EVOO (20 mL for GC-MS); EVVO (50 mL for e-nose)	R2	GC-MS SPME; e-nose	2
EVOO (20 mL for GC-MS); EVVO (50 mL for e-nose)	R3	GC-MS SPME; e-nose	3
POO (50 mL)	R1	GC-MS SPME; e-nose	4
POO (50 mL)	R2	GC-MS SPME; e-nose	5
POO (50 mL)	R3	GC-MS SPME; e-nose	6
OO (50 mL)	R1	GC-MS SPME; e-nose	7
OO (50 mL)	R2	GC-MS SPME; e-nose	8
OO (50 mL)	R3	GC-MS SPME; e-nose	9
50% adulteration: (50 mL) EVOO + (50 mL) POO	R1	e-nose	10
50% adulteration: (50 mL) EVOO + (50 mL) POO	R2	e-nose	11
50% adulteration: (50 mL) EVOO + (50 mL) POO	R3	e-nose	12
50% adulteration: (50 mL) EVOO + (50 mL) OO	R1	e-nose	13
50% adulteration: (50 mL) EVOO + (50 mL) OO	R2	e-nose	14
50% adulteration: (50 mL) EVOO + (50 mL) OO	R3	e-nose	15
50% adulteration: (50 mL) POO + (50 mL) OO	R1	e-nose	16
50% adulteration: (50 mL) POO + (50 mL) OO	R2	e-nose	17
50% adulteration: (50 mL) POO + (50 mL) OO	R3	e-nose	18
30% adulteration: (70 mL) EVOO + (30 mL) OO	R1	e-nose	19
30% adulteration: (70 mL) EVOO + (30 mL) OO	R2	e-nose	20
30% adulteration: (70 mL) EVOO + (30 mL) OO	R3	e-nose	21
30% adulteration: (30 mL) POO + (70 mL) OO	R1	e-nose	22
30% adulteration: (30 mL) POO + (70 mL) OO	R2	e-nose	23
30% adulteration: (30 mL) POO + (70 mL) OO	R3	e-nose	24
10% adulteration: (10 mL) POO + (90 mL) EVVOO	R1	e-nose	25
10% adulteration: (10 mL) POO + (90 mL) EVVOO	R2	e-nose	26
10% adulteration: (10 mL) POO + (90 mL) EVVOO	R3	e-nose	27
10% adulteration: (10 mL) POO + (90 mL) OO	R1	e-nose	28
10% adulteration: (10 mL) POO + (90 mL) OO	R2	e-nose	29
10% adulteration: (10 mL) POO + (90 mL) OO	R3	e-nose	30

**Table 2:** Sensors used in the device.

Sensor	Manufacturer	Type	Signal Measured
SHT40	Sensirion	Temperature/ Humidity	Temperature (°C), Relative humidity (%)
ENS160	Sensirion	MOX	Eco_2_ (ppm), TVOCs (ppm), Air quality Index (AQI), Four resistive elements (Ohms)
STC31-R3	Sensirion	CO_2_ sensor	CO_2_ (% vol)
	data	data	data

**Table 3:** Volatile compounds detected by GC–MS SPME for each oil. Each compound is presented in terms of abundance (a dimensional variable) represented the average of three biological replicas.

			OO	EVOO	POO	
			Code: 25-L-CR051	Code: 25-L-CR052	Code: 25-L-CR050	
RT	Volatile Compounds	CAS	Absolute Area	Absolute Area	Absolute Area	Description
5.76	Heptane	142-82-5	nd *	nd	9.66 × 10^5^	Linear saturated hydrocarbon composed of a straight chain of seven carbon atoms fully saturated with hydrogen [24].
7.82	n-Octane	111-65-9	1.50 × 10^7^	1.61 × 10^7^	2.75 × 10^6^	Single chain of eight carbon atoms bonded to eighteen hydrogen atoms [24].
8.52	Cyclohexane, 1,3-dimethyl-, cis-	638-04-0	nd	nd	1.26 × 10^6^	Chemical compound; six-carbon cycloalkane with methyl groups at positions 1 and 3. Exists as two geometric isomers: cis and trans [25].
9.52	Octene	111-66-0	1.11 × 10^6^	1.22 × 10^6^	nd	Linear alpha-olefin with double bond at position 1. Industrially produced from ethylene; used as comonomer in polyethylene and in hydroformylation to produce linear aldehydes [26].
11.83	Ethylcyclohexane	1678-91-7	nd	nd	1.20 × 10^6^	Saturated hydrocarbon: ethyl group bound to a cyclohexane ring. Found in petroleum as a naphthene; produced by hydrogenation of ethylbenzene or hydrodeoxygenation of lignin [27].
12.20	n-Nonane	111-84-2	nd	nd	2.50 × 10^5^	Straight-chain alkane; colorless liquid with sharp odor. Volatile oil component and plant metabolite; insoluble in water. Found in various plant species [28].
12.34	Acetic acid, ethyl ester, Ethyl acetate	141-78-6	8.59 × 10^6^	2.51 × 10^6^	3.90 × 10^5^	Sweet-smelling, colorless, flammable ester of ethanol and acetic acid. Widely used as a solvent in paints, nail polish, decaffeination, perfumes, and wine; also used for insect collection [29].
12.82	Acetic acid, hydroxy-(Glycolic acid)	79-14-1	1.41 × 10^7^	1.52 × 10^7^	1.27 × 10^7^	Functions as a metabolite and keratolytic agent. Used in cosmetics and dermatology; safe up to 10% (pH ≥ 3.5) in consumer products [30].
13.79	Pentanal	110-62-3	9.87 × 10^5^	1.06 × 10^6^	nd	Alkyl aldehyde; colorless volatile liquid with fruity, nutty odor. Produced via hydroformylation of butene; used in fragrance synthesis and as intermediate for plasticizers [31].
14.07	butanal, 3-methyl	590-86-3	8.20 × 10^5^	9.58 × 10^5^	nd	Branched aldehyde with a methyl group at position 3; volatile compound found in olives. Acts as flavoring agent, plant metabolite, and product of yeast metabolism [32].
15.70	Hexane, 1-methoxy-(Hexyl methyl ether)	4747-07-3	1.57 × 10^7^	1.76 × 10^7^	nd	Colorless liquid with characteristic odor. Contains a methyl group bonded to a hexane chain via oxygen; primarily used as a solvent [30].
16.70	3-buten-1-ol	627-27-0	8.32 × 10^5^	1.27 × 10^6^	nd	Organic compound belonging to the class of unsaturated alcohols. In the food sector, it can be detected as a volatile compound in certain vegetable oils [30].
17.85	3-pentanone	96-22-0	3.56 × 10^6^	1.89 × 10^6^	nd	Also known as diethyl ketone, is a simple symmetrical dialkyl ketone, with an odor like that of acetone [33].
17.95	3-methylbutanal	590-86-3	1.03 × 10^6^	8.49 × 10^5^	nd	Aldehyde, a colorless liquid and found in low concentrations in many types of food. Commercially it is used as a reagent to produce pharmaceuticals, perfumes and pesticides [34].
18.28	Decane	124-18-5	3.28 × 10^6^	3.06 × 10^6^	1.60 × 10^6^	Linear molecule of 10 carbons with a non-defined scent found in olive oil [30].
19.31	3-Ethyl-1,5-octadiene (c,t)	105-54-4	3.72 × 10^6^	6.72 × 10^6^	nd	3-ethyl-1,5-octadiene is an alkadiene that is 1,5-octadiene substituted by an ethyl group at position 3. Has a non-defined scent [3].
19.55	Methyl 3(Z)-Hexenyl Ether	70220-06-3	8.87 × 10^6^	7.83 × 106	nd	Fragrance and flavoring agent with green, fruity, slightly floral scent; methyl ether of (Z)-3-hexen-1-ol; used in flavors, fragrances, and potentially in coatings [35].
20.18	alfa-pinene	80-56-0	4.41 × 10^5^	nd	nd	Bicyclic monoterpene, a volatile organic compound commonly found in essential oils from conifers and various aromatic plants. It exhibits anti-inflammatory, antimicrobial, antioxidant, and bronchodilator properties, making it useful in traditional and pharmaceutical applications [36].
21.48	Ethyl Butanoate	105-54-4	1.03 × 10^6^	3.34 × 10^5^	nd	It is soluble in propylene glycol, paraffin oil, and kerosene. It has a fruity odor and is a key ingredient used as a flavor enhancer in processed orange juices. It also occurs naturally in many fruits, albeit at lower concentrations [37].
22.42	Butanoic acid, 2-methyl-, ethyl ester	7452-79-1	1.73 × 10^6^	3.44 × 10^5^	nd	Also known as ethyl 2-methylbutyrate. It is a fruity-scented volatile ester, used as a flavoring agent and naturally found in wines, strawberries, blueberries, apples and olive [38].
23.43	Butanoic acid, 3-methyl-, ethyl ester	108-64-5	4.33 × 10^5^	nd	nd	It has a fruity odor and flavor and is used in perfumery and as a food additive [39].
24.44	Hexanal	66-25-1	8.60 × 10^6^	1.12 × 10^7^	2.11 × 10^6^	Also called hexanaldehyde or caproaldehyde, it is an alkyl aldehyde. Its scent resembles freshly cut grass, with a powerful, penetrating characteristic fruity odor and taste. It occurs naturally and contributes to the flavor in green peas [40].
24.65	1-Propanol, 2-methyl-	78-83-1	2.21 × 10^6^	1.31 × 10^6^	nd	Also called isobutanol. It is produced by the carbonylation of propylene. Has ethereal, winey and cortex notes [41].
26.74	Isoamyl acetate	123-92-2	2.87 × 10^6^	1.10 × 10^6^	nd	Colorless liquid, slightly soluble in water, highly soluble in organic solvents. Strong banana-like odor; used as food flavoring. Naturally from bananas or synthetically produced; also, a bee alarm pheromone [42].
27.20	methyl laureate	111-82-0	5.01 × 10^5^	5.61 × 10^5^	nd	Fatty acid methyl ester of lauric acid; occurs in olive, fruits (e.g., grape, melon, pineapple), cheeses, wines, and spirits. Used as a flavoring agent; classified as a fatty acid ester [43].
27.44	2-pentenal	623-36-9	6.24 × 10^5^	6.81 × 10^5^	nd	Aldehyde found in cigarette smoke, virgin olive oil, and milk. It has a role as a plant metabolite [44].
28.72	1-Penten-3-ol	616-25-1	1.66 × 10^6^	1.80 × 10^6^	nd	Alcohol with pungent horseradish-like odor and tropical notes when diluted; used to enhance green, cucumber, melon, berry, and vegetable accords in fragrances [44].
30.33	Dodecane	112-40-3	3.50 × 10^6^	2.26 × 10^6^	1.29 × 10^6^	Linear branched molecule consisting of decane with 12 carbon atoms. It is a clear colorless liquid isolated from the essential oils of various plants including Zingiber officinale (ginger). It has a role as a plant metabolite is a natural product found in Erucaria microcarpa, with a balsamic scent found in olive oil [45].
30.50	Heptanal	111-71-7	1.28 × 10^6^	1.67 × 10^6^	3.60 × 10^5^	Aliphatic aldehyde; colorless liquid with strong fruity odor. Naturally found in ylang-ylang, clary sage, lemon, bitter orange oils, and in olives at low levels [46].
31.18	Limonene	138-86-3	8.53 × 10^5^	9.85 × 10^5^	7.20 × 10^5^	Limonene is a volatile hydrocarbon, a cycloolefin classified as a cyclic monoterpene, lemon-like odor that can be found in the rind of citrus fruits [47].
31.34	Isoamyl alcohol	123-51-3	1.12 × 10^7^	3.88 × 10^6^	nd	Isomeric alcohol: natural ester used in banana oil and as flavoring, also present in black truffle aroma. By-product of cereal fermentation, found in alcoholic beverages; component of hornet alarm pheromone [48].
32.55	2-hexenal	505-57-7	4.98 × 10^7^	3.06 × 10^7^	nd	2-Hexenal is a chemical compound of the aldehyde group. Imparts fresh, green, and natural top note in fruity floral types. Apple, berry, and other fruit flavors. Also, citrus flavors, especially orange juice [41].
32.65	3,5-dimethyl-4-aza-4-heptene	38836-40-7	8.56 × 10^7^	1.32 × 10^8^	nd	Heterocyclic compound containing a nitrogen atom as part of a seven-membered ring, with two methyl groups attached to the carbon atoms at positions 3 and 5, and an ethyl group and a methyl group attached to the carbon at position 4 [48].
33.65	1-Pentanol	71-41-0	7.32 × 10^5^	2.58 × 10^5^	nd	It is an alcohol with five carbon atoms. Pungent, fermented, bready, yeasty, fusel, winey and solvent-like smell [41].
33.94	Trans-β-Ocimene	3779-61-1	6.83 × 10^6^	7.88 × 10^6^	nd	β-Ocimene is trans-3,7-dimethyl-1,3,6-octatriene. Exists in two stereoisomeric forms, cis and trans, with respect to the central double bond. The ocimenes are often found naturally as mixtures of the various forms. Complex note, mainly herbal lavender with green citrus, metallic and mango nuances [49].
34.69	Styrene (Ethenylbenzene)	100-42-5	2.11 × 10^6^	4.40 × 10^5^	nd	Aromatic hydrocarbon. The vinyl group attached to the aromatic ring is highly reactive, as the ring can delocalize charges and unpaired electrons to the ortho and para positions through various resonance forms [50].
35.04	n-hexyl acetate	142-92-7	1.15 × 10^7^	1.32 × 10^7^	nd	Hexyl acetate is the acetate ester of hexan-1-ol. Green fruity note reminiscent of apple, pear [51].
36.11	Octanal	124-13-0	9.11 × 10^5^	1.11 × 10^6^	nd	Colorless fragrant liquid with fruity odor; naturally in citrus and olive oils. Used in perfumes and as a flavoring in food industry [52].
36.21	3-hydroxy-2-butanone	513-86-0	2.05 × 10^6^	8.07 × 10^5^	nd	Chemical used in food flavoring and fragrances; intermediate in microbial butanediol cycle; also serves as an aroma carrier in flavors and essences [52].
36.74	(E)-4,8-Dimethyl-1,3,7-nonatriene	19945-61-0	5.27 × 10^6^	5.85 × 10^6^	nd	Alkatriene consisting of 4,8-dimethylnonane having the three double bonds in the 1-, 3- and 7-positions [53].
37.41	3-Hexen-1-ol, acetate, (Z)-	3681-71-8	9.39 × 10^7^	1.34 × 10^8^	nd	Acetate ester from acetic acid and (Z)-hex-3-en-1-ol; metabolite with green, fruity aroma; found in tea, olive, and other plants [54].
38.13	2-Heptenal, (E)-	18829-55-5	2.57 × 10^6^	2.83 × 10^6^	nd	Monounsaturated fatty aldehyde with a green, fatty aroma; found mainly in pomelo peel, soybean oil, and pulses. Acts as a plant metabolite, food flavoring, and uremic toxin [19].
38.59	6-methyl-5-hepten-2-one	110-93-0	1.08 × 10^6^	1.09 × 10^6^	nd	Unsaturated methylated ketone; colorless liquid with citrus, fruity odor. Found as a mosquito attractant [55].
38.85	1-Hexanol	111-27-3	3.40 × 10^7^	2.91 × 10^7^	nd	It is an organic alcohol with a six-carbon chain. Smells pungent, ethereal, fuel oil, fruity and alcoholic, sweet with a green top note [56].
40.55	3-Hexen-1-ol, (Z)-	928-96-1	7.56 × 10^7^	8.07 × 10^7^	nd	Colorless oily liquid with intense grassy-green odor; produced by most plants as insect attractant. Key aroma compound in flavors and perfumes; used in fruit and vegetable notes [57].
41.29	Nonanal	124-19-6	9.32 × 10^6^	1.37 × 10^7^	9.78 × 10^5^	It is a formally saturated fatty aldehyde resulting from the reduction of the carboxyl group of nonanoic acid. Waxy, rose and orange peel [41].
41.50	2-Hexen-1-ol, (E)-	928-95-0	1.59 × 10^7^	8.83 × 10^6^	nd	Primary allylic alcohol derived from 2-hexene; acts as a plant metabolite. Classified as an alkenyl and allylic alcohol [58].
43.75	Acetic acid	64-19-7	3.29 × 10^7^	6.51 × 10^6^	6.72 × 10^5^	Colorless, acidic liquid; main component of vinegar. Widely used in food, chemical industry, and as acidity regulator. Central to metabolism (acetyl group) [59].
44.88	trans,trans-2,4-heptadienal	4313-03-5	1.40 × 10^6^	1.02 × 10^6^	nd	Heptadienal with double bonds at positions 2 and 4 (E,E-isomer); used as a flavoring agent [60].
46.06	α-copaene	3856-25-5	2.30 × 10^6^	1.68 × 10^6^	nd	It is an oily liquid hydrocarbon found in several plants that produce essential oils. Scents reminiscent of honey, spicy or woody notes [61].
47.59	2,3-Butanediol	513-85-9	1.72 × 10^6^	8.12 × 10^5^	nd	Organic compound; colorless vic-diol liquid. Occurs naturally in olive oil, cocoa butter, sweet corn, and rotten mussels. Used in plastics, pesticides, and GC carbonyl compound resolution [62].
47.73	α-terpinolene	586-62-9	7.31 × 10^5^	7.29 × 10^5^	nd	Natural terpene found in lilac, sage, rosemary, nutmeg, conifers, olive, and tea tree oil. Colorless to pale yellow liquid with woody, citrus-like odor; slightly bitter at high concentrations [63].
47.93	Benzaldehyde	100-52-7	1.16 × 10^6^	1.05 × 10^6^	4.73 × 10^5^	Aromatic aldehyde; colorless volatile liquid with characteristic bitter almond odor. Naturally occurs in apricot, cherry, and almond seeds (as amygdalin precursor) [64].
48.17	n-Octanol	111-87-5	1.75 × 10^6^	2.19 × 10^6^	nd	Eight-carbon alcohol; colorless liquid with characteristic odor. Hydrophobic and water-immiscible; used to determine partition coefficients of chemicals [64].
51.32	Butanoic acid	107-92-6	1.06 × 10^6^	2.03 × 10^5^	nd	Carboxylic acid found esterified in natural fats and released during fat rancidification (e.g., in butter, aged cheeses, olive). Has a pungent odor at high concentration; contributes to characteristic aroma of fermented dairy at low levels. Formed via butyric fermentation of sugars and used in the synthesis of flavor and fragrance esters [65].
51.99	Benzoic acid, methyl ester	93-58-3	1.32 × 10^6^	1.83 × 10^6^	nd	Methyl ester of benzoic acid; colorless liquid with pleasant floral odor. Found naturally in some plants; used in perfumes and as a scent marker in canine training. Poorly soluble in water, well soluble in organic solvents [66].
52.48	(E)-2-Decenal	3913-81-3	2.90 × 10^6^	3.05 × 10^6^	nd	Oily aldehyde with strong waxy odor; occurs in coriander, meats, fruits, and various foods. Used as flavoring agent and also acts as pheromone [67].
56.09	Farnesene	502-61-4	7.92 × 10^6^	1.04 × 10^7^	nd	Group of sesquiterpene isomers, including α- and β-farnesene; differ by double bond position. Found in green apple peel, cannabis, ginger, hop, and other plants. Acts as an insect alarm pheromone (e.g., aphids) and contributes to fruity, woody, and citrus aromas [68].
58.32	Benzoic acid, 2-hydroxy-, methyl ester	119-36-8	1.50 × 10^6^	1.97 × 10^6^	nd	Also known as methyl salicylate; colorless liquid with characteristic odor. Found in wintergreen oil; used in flavors, fragrances, and as a counterirritant in topical medications [69].
61.44	Benzenemethanol	100-51-6	1.22 × 10^6^	8.37 × 10^5^	nd	Aromatic alcohol; clear, colorless liquid with pleasant odor. Used as solvent, antioxidant, fragrance, and chemical intermediate; may cause irritation on contact [70].
62.78	phenylethyl alcohol	60-12-8	4.50 × 10^6^	2.12 × 10^6^	nd	Aromatic alcohol with rose-like scent; naturally found in rose, peppermint, hyacinth, and orange blossom. Used in perfumes, soaps, and as antimicrobial agent; slightly soluble in water [71].

* nd = ‘not detected’, the compound was not detected in any of the three biological replicas or was found only in one of them.

## Data Availability

The original contributions presented in this study are included in the article. Further inquiries can be directed to the corresponding authors.
